# Sick-listing adherence: a register study of 1.4 million episodes of sickness benefit 2010–2013 in Sweden

**DOI:** 10.1186/s12889-015-1741-2

**Published:** 2015-04-14

**Authors:** Ola Leijon, Malin Josephson, Niklas Österlund

**Affiliations:** Swedish Social Insurance Inspectorate, Box 202, SE-101 24 Stockholm, Sweden; Unit of Occupational Medicine, Institute of Environmental Medicine, Karolinska Institutet, SE-171 77 Stockholm, Sweden; Section of Occupational and Environmental Medicine, Department of Medical Sciences, Uppsala University, SE-751 85 Uppsala, Sweden

**Keywords:** Sickness certification, Sick-listing, Adherence, Compliance, Sickness benefit, Return-to-work, Social insurance medicine, Gender, Register study, Sweden

## Abstract

**Background:**

This register study aims to increase the knowledge on how common it is that sickness benefit recipients are sick-listed for as long as their physician prescribes in their medical sickness certificate, i.e. sick-listing adherence, or wholly/partly bring return-to-work (RTW) forward, i.e. early RTW.

**Methods:**

The unit for analysis was an episode of 100% sickness benefit, commenced between 1 January 2010 and 31 December 2013. Completed episodes of sickness benefit and full or partial early RTW was analysed by comparing the prescribed length of sick leave in medical sickness certificates and benefit days disbursed by the sickness insurance system. Probability for a full and partial early RTW was estimated with hazard ratio (HR) using the Cox proportional hazard model.

**Results:**

In total, about 1.4 million episodes of sickness benefit (60% women) were included in the study. The overall sick-listing adherence was 84% for women and 82% for men during the first year of sick leave. Adherence varied between 82 and 87% among women and between 79 and 86% among men with regard to ICD-10 diagnosis chapter. The probability of an early RTW varied between diagnosis chapters, where mental disorders was associated with a lower probability of a full early RTW among women and men (HR 0.52 and HR 0.47) as well as a partial early RTW (HR 0.51 and HR 0.46). Younger age (16–29 years), high educational level and high income was associated with a higher probability of an early RTW, while older age (≥50 years), not native-born, low educational level, unemployment and parental leave were associated with a lower probability.

**Conclusion:**

The study demonstrates that sick-listing adherence is relatively high. Probability of an early RTW differs with regard to diagnosis chapter, demographic, socioeconomic and labour market characteristics of the sickness benefit recipients. Interventions intended to improve the sick-listing process, and to affect the length and degree of sick leave in certain target groups, should include measures targeted at physicians’ sick-listing practices. Policies and economic incentives aimed at promoting RTW need to focus on individuals’ residual capacity for work.

## Background

The same type of medical, individual and social factors seems to influence both an individual’s steps and decision to be/not to be on sick leave and decision to terminate sick leave and return to work [1]. For example, factors such as diagnosis, gender, age, educational level, family and financial situation, nature of work and involvement of the employer [2-6]. Personal norms (commitment and work ethics), access to health care and rehabilitation, economic incentives, legislation and restrictions in the sickness insurance systems are also important factors for the decision [7-9]. Several studies have also shown that physicians play a significant role in their patients’ decision to take sick leave and the length of sick leave [10-15]. However, in-depth knowledge about how common it is that sickness benefit recipients follow their physicians’ prescription for sick-leave duration, i.e. sick-listing adherence, or bring return-to-work forward (RTW), i.e. early RTW, is still lacking.

In 2003, the World Health Organization (WHO) adopted the following definition of adherence to long-term therapy: ‘the extent to which a person’s behaviour – taking medication, following a diet, and/or executing lifestyle changes, corresponds with agreed recommendations from a health care provider’ [16]. This definition implies that adherence encompasses health-related behaviours and self-management that extend beyond taking prescribed pharmaceuticals. Strong emphasis was placed on the need to differentiate adherence from compliance, where the main difference is that adherence requires the patient’s involvement and agreement with the recommendations. The WHO definition of adherence may well be applied for sick-listing. Literature reviews have estimated that, in developed Western countries, poor adherence is expected in 30–50% of all patients, irrespective of disease, prognosis or setting [16-19]. Meta-analyses have demonstrated that the objective severity of disease conditions and patients’ awareness of this severity [20], as well as physician–patient communication [21], can predict patient adherence.

A new national register, that comprises the majority of medical sickness certificates issued by physicians in Sweden, makes it possible to conduct more thorough and comprehensive studies of the sickness certification process and related questions. The present study aims to increase knowledge on how common it is that recipients of 100% sickness benefit are sick-listed for the length of time physician prescribes in their sickness certificate or wholly/partly bring RTW forward. The research questions are: (i) what is level of sick-listing adherence, and (ii) does sick-listing adherence and the probability of an early RTW vary with regard to disease, patient characteristics and socio-economic factors.

### The Swedish sickness insurance system in brief

In Sweden, sick leave during the first seven days – including one qualifying day without economic reimbursement – is, with some exceptions, self-certified. Sick pay is covered by the employer from day 2 to 14. Thereafter, a person who still has reduced work ability due to disease may apply for sickness benefit from the Swedish Social Insurance Agency (SSIA). A medical sickness certificate issued by a physician is needed for such an application. In Sweden, all physicians, regardless of medical specialty, may issue a sickness certificate. The certificate should specify, among other data, patient’s diagnosis (ICD-10 code), assessment of the patient’s functional impairment and activity limitation, and recommended length and degree (100%, 75%, 50% or 25%) of sick leave. A sickness certificate does not automatically give entitlement to benefit, but constitutes one of the most important bases for the SSIA’s evaluation and decision. A new certificate is needed for prolonged sick leave. According to the Swedish National Board of Health and Welfare, sick-listing should be an integrated part of medical care and treatment, and, thus monitored by the physician that prescribes sick leave or by other health care professionals.

Since July 2008, sickness benefit has been restricted to 1 year, although it can be prolonged under certain circumstances such as severe illness. Another amendment is specific time limits, where the assessment of work ability gradually becomes stricter over time; during the first 90 days of sick leave the assessment is based on whether the person is able to do his/her regular job, during day 91–180 whether the person is able to do any job for their employer, and after that point whether the person is able to do any job the labour market has to offer. The work ability of unemployed persons is assessed in relation to jobs offered by the ordinary labour market as of the first day of sick leave [22].

## Methods

The study investigated adherence with regard to three of the five factors proposed by the WHO that may affect adherence [16]: ‘disease’, ‘patient characteristics’ and ‘socio-economic factors’ (not factors related to ‘health care system/provider–patient relationship’ or ‘other treatment than sick-listing’). Register data from the Swedish *Medical Sickness Certificates Register (MSCR)* and the database *Micro-Data for Analysis of the Social Insurance System (MiDAS database)* was utilised for the analyses. The MSCR is a national register that store data from medical sickness certificates received by the SSIA. The register contains approximately 80% of all certificates issued in Sweden during the period 1 January 2010–31 December 2013. The MiDAS database contains information on all continuous episodes of payment of sickness benefit from the SSIA, i.e. from day 15 in an episode of sick leave. The two registers are administered by the SSIA, and data in the registers were linked through a unique case number for each episode of sickness benefit.

### Study population

The study population were individuals with a new episode of sickness benefit, i.e. recipients of sickness benefit from the SSIA from day 15 in a sick leave spell, that: 1) applied for and were granted 100% sickness benefit during the period 1 January 2010–31 December 2013, and 2) for which information was available about the prescribed length of sick leave in the MSCR. A suspended payment of sickness benefit of more than five days was considered a new episode.

*Exclusion criteria* were: 1) sickness benefit recipients with a diagnosis related to pregnancy, childbirth and the puerperium, 2) episodes of partially granted – 25%, 50% or 75% – sickness benefit at the beginning of a period of sick leave, 3) episodes where the SSIA’s decision was to retract sickness benefit during the first year of sick leave, and 4) sickness benefit recipients that died during the first year of sick leave. Table 1 shows that approximately 84% of all episodes of sickness benefit in Sweden 2010–2013 remained after exclusion.

**Table 1:** **Episodes of sickness benefit commenced 1 January 2010–31 December 2013 in Sweden, and episodes included in the study**

	**Women**		**Men**		**All**	
	***n***	**(%)**	***n***	**(%)**	***n***	**(%)**
Episodes of sickness benefit^1^	1,054,730	(100)	621,940	(100)	1,676,670	(100)
*Excluded:*						
– women sick-listed for pregnancy, childbirth and the puerperium	83,323		n.a.		83,323	
– granted partial (25%, 50% or 75%) sickness benefit at the beginning of an episode	120,717		45,778		166,495	
– retracted sickness benefit during the first year of sick leave^2^	7,257		5,953		13,210	
– deceased during the first year of sick leave	3,166		4,162		7,328	
Remaining study population	840,267	(80)	566,047	(91)	1,406,314	(84)
Included in the analyses of full early RTW^3^	664,998		442,329		1,107,327	
Included in the analyses of partial early RTW^4^	795,935		540,364		1,336,299	

^1^ Total number of episodes of sickness benefit (100%, 75%, 50% or 25% of sick leave >14 days) commenced 1 January 2010–31 December 2013 in Sweden.

^2^ Number of episodes with retraction of sickness benefit during the first year of sick leave after a decision by the Swedish Social Insurance Agency, commonly after 180 days of sick leave.

^3^ Episodes of sickness benefit shorter than 365 days without information about prescribed length of sick leave in the Medical Sickness Certificates Register (MSCR) were omitted from the analysis.

^4^ Episodes of sickness benefit with reduced benefit during the first year of sick leave without information about prescribed length of sick leave at the time of reduced benefit were omitted from the analysis.

Comparisons of data in the MSCR and MiDAS database showed that episodes of sickness benefit <365 days with information on prescribed sick leave length in the MSCR (included) were, on average, shorter than the corresponding episodes lacking such information in the MSCR (not included); 62 days compared to 70 days (not shown in table).

### Sick-listing adherence and early return-to-work

Sick-listing adherence was defined as full compatibility between prescribed length of sick leave in a medical sickness certificate and disbursed days of sickness benefit during the first year of sick leave. This means that the sick-listing adherence studied included only commenced episodes of sickness benefit, i.e. sick-leave days 15–364.

Non-compatibility between prescribed length of sick leave in a sickness certificate and disbursed days of sickness benefit denotes early RTW. Full early RTW was defined as full termination of an episode of sickness benefit prior to the prescribed length of sick leave in a sickness certificate. Partial early RTW was defined as partial termination of an episode of sickness benefit (from 100% to 75%, 50% or 25% sickness benefit) prior to the prescribed length and degree of sick leave in a sickness certificate. In available register data, there was no information on what the sickness benefit recipients returned to after a sick leave period. Thus, a return to previous or new work, studies or unemployment were all considered to be RTW, i.e. the project uses a broader definition of RTW than is usually the case.

### Covariates

For each episode, data about conditions at the start of, and the year before an episode of sickness benefit was retrieved from the MiDAS database. Data at the start of an episode of sickness benefit concerned: primary diagnosis (ICD-10 chapter), gender (man/women), age (16–29/30–39/40–49/50–59/≥60 years), country of birth (Sweden/not Sweden), educational level (compulsory/secondary/tertiary education), and family situation with regard to having children 0–6 years (no/yes) and type of employment (permanently employed/short-term employed/self-employed/unemployed/ parental leave/student). Data on the year before an episode of sickness benefit concerned: employment status (working/not working, has income/not working, no income/missing data, unknown), employment sector (private company/state/county council/municipality/state- or municipality-owned company/other organisation), type of occupation (10 categories according to Swedish Standard Classification of Occupations (SSYK1)) [23] and income in SEK (<100,000/100,000–199,999/200,000–299,999/300,000–399,999/≥400,000).

### Statistical analyses

Data about medical sickness certificates, from the MSCR, was linked to the episodes of sickness benefit, from the MiDAS database, included in the study. Sick-listing adherence was analysed by comparing the last day with a valid sickness certificate with the last day with sickness benefit in a sick-leave spell. The proportion (%) of episodes with full agreement, i.e. same last day with a sickness certificate and sickness benefit, denoted the extent of sick-listing adherence.

The probability of a full early RTW or partial early RTW, respectively, was analysed with the Cox proportional hazard model. All covariates listed above were included in these analyses. Statistical significance of the hazard ratios (HR) were estimated with robust standard error, where *p* <0.05 was considered statistically significant. In the analysis of the probability of a full early RTW, episodes of sickness benefit shorter than 365 days without information about prescribed length of sick leave in the MSCR were not included in the analysis (Table 1). In the analysis of the probability of partial early RTW, episodes of sickness benefit with reduced benefit during the first year of sick leave without information about prescribed length of sick leave at the time of reduced benefit were not included in the analysis (Table 1). Of the apporximately 1.4 million remaining episodes of sickness benefit included in the study, 79% were included in the analysis of full RTW and 95% were included in the analysis of partial RTW (Table 1). Graphs of the conditional probability of early RTW during each week (3–52) among employed persons, i.e. the number of individuals who fully or partially terminate an episode of sickness benefit during a week divided by the number of individuals with an ongoing episode in the beginning of each week, is also presented. In all these analyses data was censored after 364 days of sick leave or as of 30 April 2014 for episodes that were ongoing at that time.

The analyses were conducted with Statistical Analysis Software, SAS Enterprise Guide 4.3. The Regional Ethics Committee in Stockholm, Sweden (Dnr 2013/608-31/5) approved the study.

## Results

### Sick-listing adherence

Table 2 display descriptive data on sick-listing adherence. Sick-listing adherence was 83% for all episodes of sickness benefit which lasted less than a year, and fairly similar for women and men (Table 2). Adherence varied between 82 and 87% among women and between 79 and 86% among men with regard to ICD-10 diagnosis chapter. Adherence was higher with older age and relatively high among those with a low educational level, without small children and not native-born. Sick-listing adherence also varied with regard to employment factors. Self-employed and municipality employees had a relatively high adherence, while those who were short-term employed, unemployed, students and state employees had a relatively low adherence. Adherence also varied with regard to occupational category. Those working in ‘elementary occupations’, ‘service workers and shop sales workers’ and ‘skilled agricultural and fishery workers’ had the highest adherence (87–88%), while those working in the ‘armed forces’, ‘professionals’ and ‘legislators, senior officials and managers’ had the lowest adherence (69–78%). Sick-listing adherence was relatively low in the highest income group (75%).

**Table 2:** **Sick-listing adherence in different groups during an episode of sickness benefit**

		**Sick-listing adherence** ^**1**^		
		**Women**	**Men**	**All**
		**%**	**%**	**%**
*All*		84	82	83
**At start of an episode of sickness benefit**				
*Primary diagnosis (ICD-10 diagnosis chapter)*				
I	Certain infectious and parasitic diseases	86	84	85
II	Neoplasms	83	80	82
IV	Endocrine, nutritional, metabolic diseases	84	83	84
V	Mental and behavioural disorders	85	83	84
VII	Diseases of the nervous system	82	80	81
IX	Diseases of the circulatory system	87	86	86
X	Diseases of the respiratory system	86	85	86
XI	Diseases of the digestive system	87	86	86
XIII	Diseases of the musculoskeletal system	83	81	82
XIV	Diseases of the genito-urinary system	85	85	85
XVIII	Symptoms and signs	85	85	85
XIX	Injury and poisoning	82	79	80
	Other diagnosis chapter	85	84	85
*Age group (years)*				
	16–29	82	80	81
	30–39	83	82	83
	40–49	84	82	83
	50–59	85	83	84
	≥60	85	83	84
*Country of birth*				
	Sweden	84	81	83
	Not Sweden	86	86	86
*Educational level*				
	Compulsory education	87	85	86
	Secondary education	86	82	84
	Tertiary education	81	78	80
*Children 0–6 years old*				
	No	84	82	83
	Yes	83	81	82
*Type of employment*				
	Permanently employed	85	82	84
	Short-term employed	78	79	78
	Self-employed	87	89	88
	Unemployed	76	77	77
	Parental leave	86	82	85
	Student	68	72	69
**The year before an episode of sickness benefit**				
*Employment status*				
	Working	85	82	84
	Not working, have income	82	82	82
	Not working, no income	76	80	78
	Missing data/unknown	71	75	73
*Employment sector*				
	Private company	84	83	83
	State	79	76	78
	County council	83	81	83
	Municipality	86	82	86
	State- or municipality-owned company	85	83	84
	Other organisation	83	81	82
*Occupation (SSYK1)* ^2^				
1	Legislators, senior officials and managers	77	78	78
2	Professionals	78	75	77
3	Technicians and associate professionals	82	77	80
4	Clerks	83	84	83
5	Service workers and shop sales workers	88	84	87
6	Skilled agricultural and fishery workers	86	87	87
7	Craft and related trades workers	86	83	83
8	Plant and machine operators, assemblers	87	84	85
9	Elementary occupations	89	86	88
0	Armed forces	72	69	69
	Missing data/unknown	79	81	80
*Income (SEK)*				
	<100 000	81	82	81
	100 000–199 999	85	83	84
	200 000–299 999	87	85	86
	300 000–399 999	82	82	82
	≥400 000	76	75	75

^1^ Sick-listing adherence is defined as full compatibility between physicians’ prescribed length of sick leave in sickness certificates and disbursed days of sickness benefit during the first year of sick leave.

^2^ Swedish Standard Classification of Occupations.

### Early return-to-work

**Figure 1:**
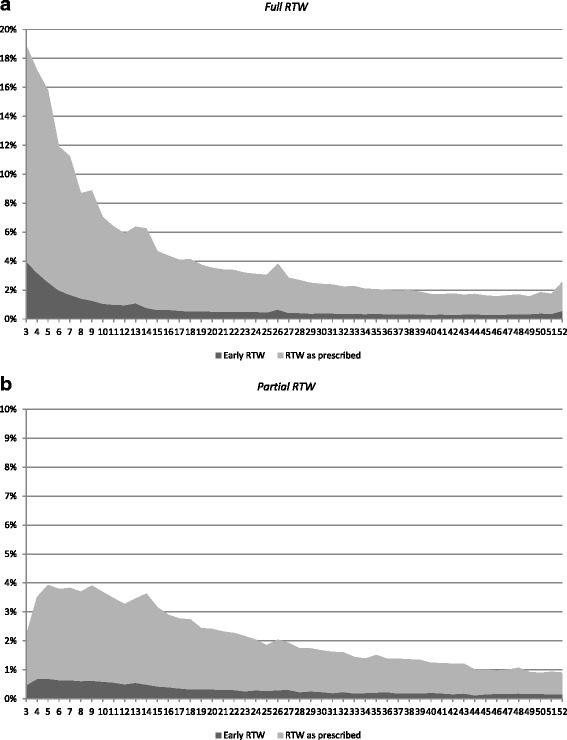
Conditional probability for return-to-work (RTW) for employed persons with a new episode of sickness benefit commenced 1 January 2010–31 December 2013, by sick leave duration week 3–52. **1a** show the likelihood of fully terminating an episode of sickness benefit (*n* = 951,112) and **1b** show the likelihood of partially terminating an episode of sickness benefit (*n* = 1,165,219), contingent on that sick leave being ongoing in the beginning of each week. Note: Employed persons with sickness benefit that ended day 180 of the sick leave were not categorised as completed, as many of the employees who terminated sick leave on that day got a retraction of sickness benefit. As a result of that they were assessed to have insufficiently reduced work ability in relation to any job the labour market had to offer.

Figure 1a displays the conditional probability of a full RTW among employed persons, i.e. the likelihood of fully terminating an episode of sickness benefit during a week, contingent on that sick leave being ongoing in the beginning of each week. The figure demonstrates that a full early RTW was more common during the first months, but also that a full early RTW occurred throughout the first year of sick leave. Additional analyses, not in the figure, show that the median number of days of a full early RTW among employed persons, in relation to the prescribed length and degree of sick leave in their sickness certificate, was 5 days (interquartile range (IQR) 12) for women and 6 days (IQR 13) for men. Among women the median number of days varied from 2 days (IQR 3) for ‘diseases in the respiratory system’ to 7 days (IQR 13) for ‘neoplasms’, and among men from 2 days (IQR 3) for ‘diseases in the respiratory system’ to 8 days (IQR 17) for ‘neoplasms’ with regard to the diagnosis in the first medical sickness certificate in an episode of sickness benefit.

Figure 1b displays a corresponding analysis of a partial RTW, i.e. the likelihood of partially terminating an episode of sickness benefit during a week contingent on that sick leave being ongoing in the beginning of each week. The figure demonstrates that a partial early RTW occurred throughout the first year of sick leave. Additional analysis, not in the figure, shows that the median number of days of a partial early RTW was 13 days (IQR 21) for women and 17 days (IQR 22) for men. Among women the median number of days varied from 5 days (IQR 9) for ‘diseases in the respiratory system’ to 22 days (IQR 44) for ‘neoplasms’, and among men from 5 days (IQR 8) for ‘diseases in the respiratory system’ to 25 days (IQR 41) for ‘neoplasms’.

#### Full early RTW

The probability of a full early RTW varied considerably between different strata (Table 3). It can be noted that ‘diseases in the respiratory system’ was associated with a higher probability of a full early RTW among both women and men (HR 1.92 and HR 1.69, respectively). Among women and men, younger age (16–29 years), high educational level and high income, and among women also short-term employment or being a student, were associated with a higher probability of a full early RTW. A relatively high probability was also seen for women and men working in the occupational categories: ‘legislators, senior officials and managers’, ‘professionals’, ‘technicians and associate professionals’, ‘clerks’, and ‘armed forces’ (HR 1.31–1.46 and HR 1.08–1.38, respectively).

**Table 3:** **The probability (hazard ratio, HR) for full early or partial early return-to-work (RTW), given the last day in the medical sickness certificate**

		**Women**			**Men**		
		**Episodes of sickness benefit**	**Full early RTW**	**Partial early RTW**	**Episodes of sickness benefit**	**Full early RTW**	**Partial early RTW**
		***n***	**HR (95% CI)**	**HR (95% CI)**	***n***	**HR (95% CI)**	**HR (95% CI)**
**At start of an episode of sickness benefit**							
*Primary diagnosis (ICD-10 diagnosis chapter)*							
I	Certain infectious and parasitic diseases	10,405	1.36 (1.29–1.44)	0.80 (0.71–0.91)	7,879	1.32 (1.25–1.41)	0.64 (0.55–0.75)
II	Neoplasms	31,154	0.62 (0.60–0.64)	0.82 (0.78–0.86)	16,109	0.62 (0.60–0.65)	0.85 (0.80–0.90)
IV	Endocrine, nutritional, metabolic diseases	16,929	1.45 (1.39–1.51)	0.74 (0.68–0.82)	6,127	1.14 (1.07–1.22)	0.51 (0.42–0.61)
V	Mental and behavioural disorders	184,028	0.52 (0.51–0.53)	0.51 (0.50–0.53)	78,431	0.47 (0.46–0.49)	0.46 (0.44–0.49)
VII	Diseases of the nervous system	28,088	1.19 (1.16–1.23)	0.70 (0.65–0.75)	13,746	1.00 (0.96–1.05)	0.62 (0.56–0.68)
IX	Diseases of the circulatory system	17,103	0.64 (0.61–0.68)	0.55 (0.51–0.60)	27,983	0.57 (0.55–0.59)	0.65 (0.61–0.69)
X	Diseases of the respiratory system	44,206	1.92 (1.86–1.97)	0.69 (0.64–0.74)	22,228	1.69 (1.63–1.76)	0.63 (0.56–0.70)
XI	Diseases of the digestive system	21,268	1.12 (1.07–1.16)	0.66 (0.60–0.72)	22,171	1.18 (1.13–1.22)	0.69 (0.63–0.76)
XIII	Diseases of the musculoskeletal system	167,612	1	1	124,945	1	1
XIV	Diseases of the genitourinary system	19,749	1.47 (1.41–1.53)	0.73 (0.66–0.79)	5,419	1.01 (0.93–1.09)	0.75 (0.64–0.88)
XVIII	Symptoms and signs	28,971	0.83 (0.80–0.86)	0.68 (0.63–0.73)	14,279	0.75 (0.72–0.79)	0.60 (0.55–0.67)
XIX	Injury and poisoning	67,000	1.21 (1.18–1.23)	1.44 (1.39–1.49)	84,980	1.20 (1.17–1.22)	1.10 (1.06–1.14)
	Other diagnosis chapter	27,255	1.06 (1.02–1.10)	0.70 (0.65–0.75)	17,136	0.93 (0.89–0.97)	0.70 (0.64–0.76)
	Missing data/unknown	1,230	0.94 (0.81–1.09)	0.37 (0.26–0.51)	896	0.80 (0.68–0.95)	0.46 (0.33–0.64)
*Age group (years)*							
	16–29	74,805	1.42 (1.39–1.46)	1.09 (1.04–1.14)	62,062	1.29 (1.26–1.32)	0.90 (0.85–0.95)
	30–39	130,212	1	1	76,761	1	1
	40–49	184,271	0.89 (0.88–0.91)	1.00 (0.96–1.04)	108,470	0.91 (0.88–0.93)	1.01 (0.97–1.06)
	50–59	190,350	0.86 (0.84–0.88)	0.91 (0.88–0.95)	126,679	0.79 (0.77–0.81)	0.88 (0.84–0.93)
	≥60	85,360	0.86 (0.84–0.88)	0.67 (0.63–0.70)	68,357	0.79 (0.77–0.81)	0.67 (0.63–0.71)
*Country of birth*							
	Sweden	564,043	1	1	369,501	1	1
	Not Sweden	100,955	0.85 (0.83–0.87)	0.67 (0.65–0.70)	72,828	0.76 (0.74–0.78)	0.59 (0.56–0.62)
*Educational level*							
	Compulsory education	84,612	0.88 (0.86–0.90)	0.81 (0.77–0.85)	96,543	0.90 (0.89–0.92)	0.85 (0.82–0.89)
	Secondary education	358,839	1	1	259,013	1	1
	Tertiary education	221,547	1.12 (1.10–1.14)	1.19 (1.15–1.23)	86,773	1.13 (1.10–1.15)	1.42 (1.36–1.47)
*Children 0–6 years old*							
	No	556,381	1	1	368,705	1	1
	Yes	108,617	1.07 (1.05–1.10)	1.06 (1.02–1.11)	73,624	1.04 (1.01–1.06)	1.04 (1.00–1.09)
*Type of employment*							
	Permanently employed	573,358	1	1	377,754	1	1
	Short-term employed	11,718	1.20 (1.15–1.25)	0.68 (0.61–0.76)	5,370	1.06 (0.99–1.13)	0.78 (0.67–0.90)
	Self-employed	11,614	0.78 (0.75–0.80)	0.32 (0.29–0.34)	22,371	0.75 (0.72–0.77)	0.20 (0.18–0.22)
	Unemployed	53,947	0.71 (0.67–0.75)	1.80 (1.67–1.94)	34,679	0.66 (0.63–0.69)	1.43 (1.34–1.53)
	Parental leave	13,743	0.85 (0.81–0.90)	0.61 (0.55–0.68)	1,973	0.85 (0.76–0.95)	0.64 (0.51–0.82)
	Student	618	1.54 (1.33–1.79)	0.64 (0.40–1.01)	182	1.19 (0.90–1.59)	0.52 (0.20–1.37)
**The year before an episode of sickness benefit**							
*Employment status*							
	Working	598,522	1	1	398,393	1	1
	Not working, have income	30,805	0.88 (0.83–0.92)	0.65 (0.59–0.72)	19,433	1.01 (0.96–1.06)	0.75 (0.66–0.85)
	Not working, no income	11,020	0.79 (0.74–0.84)	0.68 (0.59–0.79)	7,585	0.73 (0.68–0.79)	0.72 (0.60–0.87)
	Missing data/unknown	24,651	0.76 (0.72–0.80)	0.53 (0.47–0.60)	16,918	0.77 (0.73–0.82)	0.78 (0.68–0.90)
*Employment sector*							
	Private company	234,979	1.18 (1.14–1.22)	1.47 (1.40–1.55)	314,721	1.06 (1.01–1.11)	0.99 (0.91–1.08)
	State	28,233	1.03 (1.01–1.05)	1.26 (1.21–1.31)	15,064	0.86 (0.82–0.92)	1.17 (1.06–1.30)
	County council	65,131	1.02 (0.99–1.05)	1.07 (1.00–1.14)	8,773	0.99 (0.96–1.03)	1.15 (1.06–1.24)
	Municipality	240,485	1	1	36,145	1	1
	State or municipality owned company	31,107	1.03 (1.01–1.05)	1.21 (1.17–1.26)	29,721	0.88 (0.86–0.91)	1.15 (1.09–1.23)
	Other organisation	29,392	1.08 (1.05–1.12)	1.24 (1.17–1.31)	13,402	0.93 (0.89–0.98)	0.97 (0.88–1.07)
*Occupation (SSYK1)*							
1	Legislators, senior officials and managers	14,107	1.32 (1.26–1.38)	2.18 (2.04–2.32)	14,679	1.08 (1.03–1.13)	1.81 (1.67–1.97)
2	Professionals	78,673	1.39 (1.35–1.43)	1.89 (1.80–1.98)	32,249	1.28 (1.24–1.34)	1.88 (1.75–2.03)
3	Technicians and associate professionals	108,525	1.26 (1.23–1.29)	1.69 (1.62–1.76)	45,918	1.26 (1.22–1.31)	1.69 (1.58–1.81)
4	Clerks	54,999	1.31 (1.27–1.34)	1.92 (1.84–2.01)	21,919	1.10 (1.06–1.15)	1.32 (1.21–1.44)
5	Service workers and shop sales workers	236,067	1	1	37,841	1	1
6	Skilled agricultural and fishery workers	3,009	1.02 (0.93–1.13)	1.43 (1.22–1.68)	8,572	0.98 (0.91–1.05)	1.61 (1.45–1.79)
7	Craft and related trades workers	6,465	1.07 (1.00–1.15)	1.25 (1.11–1.42)	93,131	1.02 (0.99–1.05)	0.92 (0.86–0.98)
8	Plant and machine operators, assemblers	25,963	1.02 (0.99–1.07)	1.01 (0.94–1.09)	85,551	1.06 (1.03–1.10)	0.90 (0.84–0.96)
9	Elementary occupations	51,508	0.95 (0.92–0.98)	1.05 (0.99–1.12)	30,694	0.97 (0.93–1.01)	0.86 (0.79–0.95)
0	Armed forces	116	1.46 (1.03–2.08)	1.01 (0.50–2.04)	819	1.38 (1.21–1.58)	1.01 (0.74–1.38)
	Missing data/unknown	85,566	1.23 (1.19–1.29)	1.31 (1.22–1.42)	70,956	1.04 (0.99–1.08)	0.98 (0.90–1.07)
*Income (SEK)*							
	<100 000	39,613	1.09 (1.05–1.12)	0.79 (0.73–0.85)	27,289	1.03 (0.99–1.07)	0.77 (0.70–0.84)
	100 000–199 999	139,175	0.93 (0.91–0.95)	0.76 (0.73–0.79)	56,053	0.94 (0.91–0.97)	0.78 (0.73–0.82)
	200 000–299 999	312,278	1	1	141,474	1	1
	300 000–399 999	130,913	1.26 (1.24–1.28)	1.34 (1.30–1.39)	149,323	1.26 (1.24–1.29)	1.25 (1.20–1.30)
	≥400 000	43,019	1.49 (1.45–1.53)	1.83 (1.75–1.91)	68,190	1.71 (1.67–1.75)	1.72 (1.65–1.80)

A lower probability of a full early RTW among women and men sick-listed for ‘mental and behavioural disorders’ (HR 0.52 and HR 0.47, respectively) can also be noted. A relatively low probability was also seen for women and men in the age groups ≥40 years, not native-born, low educational level, self-employed, unemployed, and on parental leave.

#### Partial early RTW

The probability of a partial early RTW also varied between different strata (Table 3). Women and men sick-listed for ‘injury, poisoning and certain other consequences of external causes’, had a higher probability of a partial early RTW (HR 1.44 and HR 1.10, respectively). Among women and men, high educational level, high income, and self-employed was associated with a higher probability of a partial early RTW. In addition, a relatively high probability was seen for women and men working in the occupational categories: ‘legislators, senior officials and managers’, ‘professionals’, ‘technicians and associate professionals’, ‘clerks’ and ‘skilled agricultural and fishery workers’ (HR 1.43–2.18 and HR 1.32–1.88, respectively).

Women and men sick-listed for ‘mental and behavioural disorders’ (HR 0.51 and HR 0.46, respectively), and ‘diseases in the circulatory system’ (HR 0.55 and HR 0.65) had a lower probability of a partial early RTW. A lower probability was also noted for women and men in the age groups ≥50 years, not native-born, low educational level and low income. Women and men that were unemployed, short-term employed, students or on parental leave had a relatively low probability of a partial early RTW.

## Discussion

### Main findings

About four out of five female and male sickness benefit recipients terminated sick leave the same day their medical sickness certificate expired, thus sick-listing adherence seems to be high. The probability of termination of an episode of sickness benefit prior to the prescribed length of sick leave in the medical sickness certificate varied between ICD-10 diagnosis chapters. Sickness benefit recipients due to ‘mental and behavioural disorders’ had a lower probability of an early RTW than individuals sick-listed due to somatic diagnoses. High educational level, high income and occupational categories requiring a high level of skills were associated with a higher probability of a full or partial early RTW. On the other hand, older age (≥50 years), not native-born, low educational level, unemployment and parental leave were associated with a lower probability of a full or partial early RTW. Some of the key findings in the study are discussed below.

### Sick-listing adherence is high

Sick-listing adherence seems to be higher than, for example, adherence to medication [16-19], scheduled outpatient appointments for primary care [24] and healthy life style habits [25]. However, there is largely lacking research on adherence to medical treatments that are not about medication, and we want to emphasise that it is not recommended to compare sick-listing adherence with adherence to other types of medical interventions, for example pharmaceutical treatment or recommendations about weight reduction or smoking cessation. It should also be noted that the concept of sick-listing adherence might be ambiguous depending on the perspective used to interpret results. A high adherence may indicate that the patients are doing as their physician recommends. This may be beneficial from a medical point of view, but may on the other hand lead to unnecessary long sick leave periods if the prescription of sick leave is too long or if the patient recovers earlier than expected. The latter is often discussed as a societal problem in terms excessive sick leave. A low adherence may indicate that the patient does what he or she finds most appropriate based on their own circumstances and beliefs. A low adherence may not necessarily be the best for a medical point of view, but may on the other hand be an indication of high patient autonomy and is obviously also economically beneficial from a societal point of view. In order to broaden the understanding, there is a need of both qualitative studies to disentangle the concept of sick-listing adherence and quantitative longitudinal studies to investigate the individual and societal effects of low and high adherence.

There may be several explanations for our result of a relatively high adherence to sick-listing. It has been reported that sick-listed employees often find it difficult to decide when they are ready to resume work [26]. Factors such as own personality and norms, lack of social support at the workplace, poor access to health care, and obstacles in the coordination of insurance, social and rehabilitation systems may potentially, singly or in combination, reduce the motivation to and opportunity for the individual to bring RTW forward [26]. Another explanation could be that adherence to sick-listing depends on the quality of the patient–physician interaction and communication [21]. Effective communication may contribute to patient adherence to the recommendations of the physician and/or that the physician considers more carefully the individual’s needs in determining sick leave with regard to length and degree. However, it has been shown that both patients’ and general practitioners’ ability to predict sick-leave length is rather weak [27]. It has also been reported that it is not uncommon that physicians issue sickness certificates for longer periods than is actually necessary [14]. This was to a large extent associated with frequency of problems, lack of time during the consultations, delicate interactions with patients, and the need for more competence in social insurance medicine. In addition, patient–physician communications may be hampered by the physician’s ‘dual role’: being both the patient’s advocate and a medical expert (gate keeper) for the sickness insurance system [28].

We recognise that a medical certificate does not capture all of the communication that takes place between the physician and the patient during a consultation. The communication may, for example, contain elements where the patient is encouraged to resume work when he or she feels ready for it, that is, if possible, before the sickness certificate expires. Such information is not usually in a medical certificate unless the physician has explicitly set the date for a partial RTW, in other words, when the physician has prescribed a gradual reduction of the degree of sick leave.

Sick-listing adherence was comparably low for those with short-term employment (78%). Short-term employees, without entitlement to sick pay (from the employer), often have precarious working conditions with a weaker position in the labour market than permanent employees. Therefore, they may have greater incentives for accelerating RTW and, hence, reducing the risk of not getting extended employment [29]. A recently published Finnish study, in contrast to our assumptions, found that temporary employment was associated with a slower RTW [30]. However, that study was restricted to public sector employees and work disability due to depressive disorders, while our study included all episodes of sickness benefit in Sweden regardless of employment sector and underlying diagnosis for reduced work ability.

### Early return-to-work varies between different groups of sickness benefit recipients

In many Western welfare states, different types of early RTW policies [31] and economic incentives [32] have been implemented in order to accelerate RTW. There may be diverse motives behind such initiatives, for example to reduce the economic costs of the social insurance system or to promote health among sick-listed individuals. Several studies and systematic reviews indicate the beneficial mental and physical health effects of work resumption [26,33-35]. In the UK, for example, a new policy has been introduced in order to change perceptions about what constitutes ‘fitness for work’ [36], i.e. a policy that challenges the belief that it is necessary to be completely (100%) well in order to be at work and that work normally impedes recovery. Accordingly, the policy focuses on the residual capacity for work people have while unwell [29]. In Sweden, partial sick leave is considered as a means to enhance RTW and is used relatively often towards the end of a sick-leave period. Nevertheless, the question of whether an early RTW might increase ‘sickness presenteeism’, i.e. coming into work unwell and not performing one’s role to full effectiveness [29,37] has also been discussed. Sickness presenteeism may have implications for both the employer in terms of reduced productivity and for the employee in terms of increased strain and, therefore, risks the need for extension of time to recovery. So it is still uncertain, and perhaps always will be, what the optimal length of sick leave in various disease states and in relation to different work tasks and jobs is.

Our study found that high educational level and high income was associated with a higher probability of an early RTW. The replacement level in the social insurance system as well as working conditions and work tasks may potentially and explain these results in part. A low benefit level may serve as an economic incentive for the group with a high income, because, in economic terms, they lose most from being absent from work. A Dutch study found that a low household income was a predictor for RTW in workers without comorbidity while better mental health was a predictor for RTW in workers with comorbidity [38]. In addition, persons with a high income and high educational level may often have flexible work arrangements, and opportunities for work adjustments or modification of work tasks (work autonomy), when work capacity is reduced [29,39]. This is probably true for many of the jobs in the occupational categories ‘legislators, senior officials and managers’, ‘professionals’, ‘technicians and associate professionals’ and ‘clerks’, where the sick-listed had a higher probability of a full or partial early RTW. However, further research is needed to investigate why an early RTW differs between occupational groups: if there are specific factors facilitating or hindering an early RTW in certain jobs or if in some jobs it is easier to exploit employees’ residual work capacity.

The probability of an early RTW varied with regard to sick leave diagnosis (ICD-10 chapter). Our study found that sick-listed persons due to mental disorders had a lower probability of a full or partial early RTW than individuals sick-listed due to musculoskeletal disorders or other somatic diagnoses. One reason could be that knowledge about musculoskeletal disorders, sick leave and RTW is more extensive among both the sick-listed and employers, i.e. knowledge on why and how work may have the potential to be an important part of the recovery process. Such knowledge is presumably lacking for mental disorders. The RTW process is often particularly difficult for employees’ with mental disorders due to various aspects of the severity of the individuals’ mental health problems: the duration of the problems prior to the occurrence of sick leave or seeking help, and the level of symptoms (somatisation, anxiety and depression) [40]. Moreover, the employees’ mental illness is often hidden: either because the individual is not fully aware of their symptoms or not open with his/her problems. Additionally, the workplace may not be willing or able to accommodate the employee’s problem. The employee may be worried that he/she will be discriminated against or not taken seriously when his/her superiors and colleagues know about the mental illness, and/or that the disease is factored into everything he/she do or say [41]. A hidden (or neglected) disease makes it difficult for the employer to take appropriate measures to facilitate RTW.

### Methodological considerations

This study has certain strengths and limitations that are worthwhile mentioning. A major strength of our study is that it included the majority of episodes of sickness benefit during a four-year period. A further strength is that the data were retrieved from primary sources, namely the medical sickness certificates and disbursed days of sickness benefit by the SSIA. The study is somewhat limited in that not all sickness certificates are in the MSCR. Episodes of sickness benefit that lacked information on prescribed sick-leave length in the MSCR were on average somewhat longer than those included in the study. However since adherence was rather similar between short and long episodes of sickness benefit this probably only affected the results to a lesser extent. The study is also limited due to a lack of data on any previous episodes of sickness benefit an individual may have had, and if the sickness benefit recipient was granted any other temporary benefits by the SSIA such as pregnancy benefit or parental benefit. Finally, in the study we did not have access to data on persons that did not apply for or were denied sickness benefit despite having had a sickness certificate. Therefore, we were only able to study sick-listing adherence with regard to commenced episodes of sickness benefit.

## Conclusions

The main finding of the present study is that sick-listing adherence is relatively high, i.e. most of the sick-listed returned to work the same day their sickness certificate expired. This also means that in almost one out of five episodes of sickness benefit, the sick-listed returned to work earlier than the length and degree of sick leave prescribed by their physician. Both sick-listing adherence and probability of an early RTW varied with regard to ICD-10 diagnosis chapter and between different demographic, socio-economic and labour market groups. The results clearly illustrate that the physician’s role is essential with regard to the length of an episode of sickness benefit. Interventions intended to improve the sick-listing process, and to affect the length and degree of sick leave in certain target groups, should include measures aimed at physicians’ sick-listing practices. In addition, policies and economic incentives aimed at promoting the RTW process need to focus on individuals’ residual capacity for work. Further research in this field – preferably with gender-sensitive approaches and strategies – is also needed in order to investigate causes for the difference in sick-listing adherence, work-related factors that may facilitate or hinder sick-listed persons bringing RTW forward and the employer’s role in the RTW process. Further studies are also needed to disentangle whether and how the health care system–/provider–patient relationship and treatment may affect sick-listing adherence.

## Abbreviations

HR: Hazard ratio
ISF: Inspektionen för socialförsäkringen [Swedish Social Insurance Inspectorate]
MiDAS: Micro-Data for Analysis of the Social Insurance System
MSCR: Medical Sickness Certificates Register
RTW: Return-to-work
SEK: Swedish krona, the currency of Sweden
SSIA: Swedish Social Insurance Agency
WHO: World Health Organization
